# Assessment and Management of Electrical Injuries in Adults in the Emergency Department

**DOI:** 10.7759/cureus.107162

**Published:** 2026-04-16

**Authors:** Ireland Smith, Savannah Kidd, Sharon Kim, Robert M Tennill

**Affiliations:** 1 Emergency Medicine, Southern Illinois University School of Medicine, Springfield, USA

**Keywords:** electrical burns, electrical injury, hvei, lightning, lvei

## Abstract

Electrical burns can cause a variety of clinical sequelae, including cardiac, renal, muscular, neurological, and ocular complications that require immediate monitoring and/or intervention by emergency medicine physicians to prevent further organ damage. These injuries can occur through a variety of mechanisms, including household electrical outlets, occupational exposures, and lightning strikes. The intensity of the electrical insult plays a major role in determining the severity of the resulting medical complications. Electrical injuries can lead to a wide range of subsequent complications involving almost every organ system in the body. Cardiac and respiratory complications are of utmost concern because they pose an immediate threat to a patient’s life, while other complications may include thermal burns, compartment syndrome, kidney dysfunction, seizures, and neuropathy, among other conditions. Early intervention in the ED is critical for reducing mortality and improving overall outcomes in these patients. This paper serves as a comprehensive review of the most up-to-date practices in the management of electrical burn injuries in the ED by addressing each major organ system and recommending current best practices.

## Introduction and background

Electrical injuries affect more than 30,000 individuals annually in the United States and account for approximately 4% of all burn center admissions in both adults and children [1-3]. While the majority are nonfatal, about 3% result in death, with high-voltage electrical injuries (HVEIs) responsible for roughly 40% of fatalities and lightning strikes for about 5% [1,3]. Electrical injuries disproportionately affect males and are associated with occupational exposures in high-risk professions such as electricians, line workers, and utility workers [4]. While low-voltage electrical injuries (LVEIs) are more common, HVEIs cause greater morbidity, with higher rates of amputation, longer hospital stays, and more complex systemic complications [4,5]. Pediatric electrical injuries are less frequent and, encouragingly, recent U.S. data suggest a decline in high-voltage exposures among children, likely reflecting improved electrical safety standards [6]. When pediatric injuries do occur, they are most often low-voltage exposures from household electrical outlets [1].

LVEIs are defined as electrical exposures of less than 1000 volts (V); however, most household appliances operate at below 240 V. HVEIs occur at voltages greater than 1000 V and are most commonly occupational hazards resulting from contact with power lines, electrified train systems, improper use of electricity, and a lack of awareness of electrical hazards [4,5]. Lightning strikes are included under the definition of HVEI and are estimated to involve 30,000 to 110,000 V per strike, resulting in significantly higher mortality and morbidity [6-8]. Lightning strikes can occur through a variety of mechanisms, including direct strike, contact between conductive objects and the victim, ground current reaching the victim, or electrical injury from associated explosions [8,9].

Electrical currents are measured in amperes, and the force that drives the current is measured in volts. Electrical current has two forms: direct current and alternating current. With direct current, electrons flow in one direction. This is seen in lightning, defibrillators, and pacemakers. With alternating current, electrons periodically reverse direction within a conductor. This is commonly found in homes and standard work buildings. The latter is considered more dangerous because it can cause tetanic contractions on contact, resulting in a longer duration of electrical exposure [10].

When an individual comes into contact with uncontained electrical energy, the current travels through the body along nerves, muscles, and blood vessels before exiting. These tissues sustain more substantial damage as a result of the electrical encounter than the skin, bone, and adipose tissue because of their higher conductivity [1]. The risk of tissue damage and subsequent medical complications is greater with prolonged contact with the electrical current and with higher-voltage exposure [11]. Additionally, the direction of the current pathway has also been associated with the severity of injury. Vertical pathways allow the electrical current to come into contact with the brain, heart, lungs, respiratory muscles, and uterus, resulting in more widespread injury to these organs. Horizontal pathways may involve the heart and lungs only, while a pathway limited to the lower body may cause only local tissue damage [10]. Contact with electricity can cause a variety of medical sequelae, some acutely life-threatening and others delayed and associated with reduced quality of life. In this clinical review, we attempt to consolidate the broad spectrum of medical complications of electrical injuries in adults presenting to the ED, as well as recommend best practices in management for each organ system by emergency physicians based on the current literature.

## Review

Methods

We performed a narrative review of the literature on electrical injuries, with a focus on systemic complications and ED management. Searches were conducted using PubMed through July 2025. Search terms included general descriptors (“electrical injury,” “electrical burn,” “low-voltage electrical injury (LVEI),” “high-voltage electrical injury (HVEI),” and “lightning”); organ-system complications (“cardiac arrest,” “troponin,” “arrhythmias,” “myocardial infarction,” “respiratory complications,” “inhalation injury,” “pulmonary hemorrhage,” “acute respiratory distress syndrome,” “acute kidney injury,” “renal failure,” “rhabdomyolysis,” “compartment syndrome,” “neurologic complications,” “seizures,” “stroke,” “peripheral neuropathy,” “ocular injury,” “cataract,” “auditory injury,” and “tympanic membrane”); and management and outcomes (“fluid resuscitation,” “Parkland formula,” “Brooke formula,” “advanced cardiac life support (ACLS),” “advanced trauma life support (ATLS),” and “emergency department management”).

Reference lists of relevant articles were also screened to identify additional publications. Articles were limited to English-language publications and included clinical studies, case series, review articles, and guideline statements. Studies involving both adult and pediatric populations were reviewed, although emphasis was placed on complications and management strategies relevant to adult patients presenting to the ED.

Discussion

Management of Electrical Burn Complications in the ED

Currently, there is a lack of widely accepted guidelines for treating electrical injuries; therefore, management is primarily based on current trauma and burn protocols. The Wilderness Medical Society has published a standard treatment protocol specifically for lightning injuries, which includes ATLS principles, routine ECG, head CT if there is loss of consciousness or a direct head strike, ophthalmologic evaluation, bilateral ear examinations, and burn care for superficial injury [6]. For LVEI and HVEI, the literature recommends starting with ATLS and continuing with a routine workup. This includes assessment and management of the airway, breathing, and circulation (ABCs). An initial ECG should be obtained to assess for cardiac arrhythmias. A basic laboratory workup, including a complete blood count (CBC), comprehensive metabolic panel (CMP), and baseline creatine kinase (CK), should be obtained in all patients, as some values are early indicators of complications and mortality. Patients who experience HVEI or lightning strikes should be closely monitored for complications of electrical injury, including delayed arrhythmias, respiratory distress, acute kidney injury (AKI), fluid overload, and compartment syndrome. The following sections address the most up-to-date, evidence-based recommendations for each specific system.

Cardiovascular

A common pathway for electricity to travel is through the heart along neurovascular structures, as this offers a path of least resistance for the current to exit the body. Electrical energy interferes with normal cardiac activity and causes cardiac myocyte damage, leading to tachycardia, arrhythmias, and, in severe cases, cardiac arrest [12]. Most arrhythmias occur within the first few hours after exposure to electrical current. Sinus tachycardia and premature ventricular contractions are the most common arrhythmias following electrical injury; however, more serious arrhythmias such as ventricular fibrillation, ventricular tachycardia, and asystole have also been reported. One study showed that patients with LVEI more frequently developed ventricular arrhythmias after injury, whereas those with HVEI were at higher risk of pulseless electrical activity (PEA) and asystole [13]. Of note, patients struck by lightning are at increased risk of fatal cardiac arrhythmias because of the higher voltage exposure.

First responders should ensure scene safety before performing ACLS for unresponsive individuals. For lightning victims, the American Heart Association recommends vigorous resuscitation efforts in cases of cardiac arrest, as these individuals may be more responsive to such measures [14]. Upon arrival at the ED, all victims of electrical injury should undergo an initial ECG, as this is one of the most critical components in determining the severity of cardiac injury and the need for prolonged monitoring. If there are any new abnormalities on the ECG, or if there was any loss of consciousness after the electrical injury, the patient is at higher risk of cardiac events and requires at least 24 hours of monitoring [10,15]. Likewise, if the patient has a history of arrhythmias, coronary artery disease, myocardial infarction, or HVEI, they are also at increased risk of adverse cardiac events following electrical injury and should be placed on continuous telemetry for at least 24 hours. Patients with no known cardiac disease, no loss of consciousness after electrical injury, a normal ECG on arrival at the ED, and LVEI do not require in-hospital cardiac monitoring [16-18].

Obtaining cardiac enzymes such as troponin or creatine kinase-MB (CK-MB) levels has not been shown to be helpful in cardiac risk stratification after electrical injuries, as elevations in these biomarkers may be variable following electrical insult [17]. However, cardiac biomarkers may be helpful if the patient presents with new-onset chest pain, hemodynamic instability, or ECG findings suggestive of acute coronary syndrome, as these patients may require emergent coronary angiography. Myocardial infarction after electrical injury is rare but should not be completely excluded if clinical suspicion is high and the patient has cardiovascular risk factors [19].

In summary (Table 1), an ECG on arrival at the ED is essential in patients who have sustained electrical injuries, as any abnormality may indicate cardiac damage and future risk of adverse cardiac events requiring more extensive monitoring. Across a variety of studies, troponin and CK-MB have limited utility for assessment of cardiac damage, except in patients experiencing new-onset chest pain, hemodynamic instability, or ECG findings concerning for ACS following electrical injury. Cardiac monitoring for 24 to 48 hours is advised for patients with an abnormal ECG compared with baseline, cardiac arrest due to electrical insult, loss of consciousness, or HVEI/lightning exposure.

**Table 1:** Management of cardiac complications in the ED. Table Credits: Ireland Smith, Savannah Kidd ACLS: Advanced Cardiac Life Support; CK-MB: Creatine kinase-MB; ACS: Acute coronary syndrome; HVEI: High-voltage electrical injury; LVEI: Low-voltage electrical injury.

S. No.	Management of cardiac complications
1	ACLS protocol for cardiac arrest [14]
2	Vigorous resuscitation efforts for lightning strike victims [14]
3	Initial ECG upon arrival at the ED [10,15]
4	Cardiac biomarkers, including troponin and CK-MB, are recommended only for patients experiencing chest pain, hemodynamic instability, or ECG findings concerning for ACS [17]
5	Telemetry monitoring for at least 24 hours if there is an arrhythmia on the initial ECG, loss of consciousness, a history of significant cardiac disease, or an HVEI/lightning mechanism [10,15-18]
6	Discharge if asymptomatic with a normal ECG after LVEI [16-18]

Musculoskeletal

Musculoskeletal injury is common in electrical burns, as electrical current follows the path of least resistance, favoring conductive tissues such as nerves, vessels, and muscles. In contrast, high-resistance tissues such as bone and tendons absorb more heat, resulting in greater thermal injury [20]. The passage of current can induce electroporation and electroconformational protein denaturation, disrupting cell membranes and triggering cell necrosis and apoptosis. Electroporation increases membrane permeability and causes direct myocyte lysis, contributing to myonecrosis [21-23]. The subsequent release of intracellular contents, including CK and myoglobin, predisposes patients to rhabdomyolysis, AKI, and compartment syndrome [24]. Importantly, deep tissue damage may far exceed cutaneous findings, requiring a high index of suspicion and thorough evaluation.

Muscle breakdown with subsequent rhabdomyolysis is a common consequence of electrical burns. The clinical presentation of rhabdomyolysis is variable, as the classic triad of myalgia, weakness, and dark-colored urine occurs in less than 10% of cases, and fewer than half of patients report muscle pain or weakness. Nonspecific symptoms may include fever, malaise, nausea, tachycardia, and generalized discomfort. Examination may reveal swollen, tender muscle groups [25,26]. Diagnosis is confirmed by a serum CK level greater than five times the upper limit of normal, typically >1000 IU/L [25-28]. Additional findings can include myoglobinuria, hyperkalemia, hyperphosphatemia, and evidence of AKI. Importantly, treatment should not be delayed while awaiting laboratory results if rhabdomyolysis or compartment syndrome is suspected [27,29].

Close monitoring is essential following electrical injury, as muscle damage may progress despite minimal external findings. Serum CK should be obtained on arrival to assess for rhabdomyolysis, with serial measurements used to monitor trends and guide ongoing management. Even if initial values are normal, CK should be trended for 24-48 hours, as levels may rise later in the course. CK levels typically peak within 1-3 days and remain a predictor of limb loss and mortality throughout the first week [24,30]. Admission and fluid resuscitation decisions should be guided by the overall clinical context, including renal function and evolving limb findings, rather than by a single CK threshold. Limb examinations should be performed in all patients with electrical burns every one to two hours during the first 24-48 hours to detect evolving rhabdomyolysis or compartment syndrome [31].

Compartment syndrome may develop from both thermal and non-thermal mechanisms of deep muscle injury. It results from rising pressure within a fascial compartment, compromising perfusion and tissue viability [32-34]. The hallmark is pain out of proportion to the injury, often worsened by passive stretch. Other findings include paresthesia, pallor, paralysis, poikilothermia, and pulselessness, but these are late signs [31,34]. In patients with unreliable examinations, the diagnosis may be supported by intracompartmental pressure measurement. While an absolute compartment pressure greater than 30 mmHg may warrant intervention, interpretation should be made in the context of clinical findings and perfusion status. A delta pressure (diastolic blood pressure minus compartment pressure) of less than 30 mmHg indicates impaired perfusion and is the preferred threshold for surgical decision-making, with fasciotomy warranted when this value is reached [31,34]. Surgery should be consulted early for suspected cases, and bedside fasciotomy may be performed when rapid intervention is required and transfer to the operating room is not feasible. Although any electrical burn can precipitate compartment syndrome because of deep muscle necrosis, circumferential burns are a particularly strong risk factor and should prompt close monitoring and early consideration of intervention [33,35]. Compartment syndrome can present immediately or evolve over hours and is a strong predictor of poor outcomes such as amputation [33]. Early recognition and fasciotomy are essential to prevent severe damage [31,35].

Beyond thermal tissue damage, electrical injuries can also cause significant musculoskeletal trauma because of tetanic muscle contractions, which may generate sufficient force to produce fractures and dislocations [36]. Reported injury patterns include posterior shoulder dislocation, humeral fractures, scapular fractures, distal radius fractures, and vertebral compression fractures, with posterior shoulder dislocation representing the most commonly described joint injury [37-41].

All patients with electrical injuries should undergo a thorough musculoskeletal examination, as significant injury may occur in the absence of visible entry or exit wounds. Plain radiographs are recommended for patients with localized pain or swelling, restricted range of motion, pain severity out of proportion to cutaneous findings, or examination findings suggestive of joint dislocation [40,42,43]. Point-of-care ultrasound may facilitate rapid diagnosis of shoulder dislocation in the emergency setting [44].

In high-voltage injuries, MRI may be required to evaluate the extent of deep soft tissue injury, though this is typically performed after admission [45]. Early reduction of dislocations is recommended, as delayed management may be complicated by worsening muscle spasm and a decreased likelihood of successful closed reduction [43]. Orthopedic or surgical consultation is indicated for suspected compartment syndrome, high-voltage injuries with deep tissue involvement, and cases involving fractures or dislocations [31]. Patients with uncomplicated dislocations that are successfully reduced may be appropriate for outpatient orthopedic follow-up [31,35].

Electrical burns often cause muscle injury that far exceeds visible skin damage, necessitating a high index of suspicion. Vigilant laboratory and clinical monitoring are critical for early detection of rhabdomyolysis and compartment syndrome. Prompt recognition and timely intervention, including fasciotomy when indicated, remain essential to reducing the risk of limb loss and systemic complications (Table 2).

**Table 2:** Management of musculoskeletal complications in the ED. Table Credits: Ireland Smith, Savannah Kidd CK: Creatine kinase; mmHg: Millimeters of mercury.

Management of musculoskeletal complications
Obtain a CK level on arrival at the ED; trend for 24-48 hours even if initially normal [24,30].
Admit for aggressive IV fluid resuscitation if CK is >5 times the upper limit of normal or if there is clinical concern for rhabdomyolysis [24-30].
Evaluate the limbs for circumferential burns that predispose to compartment syndrome [33,35].
Perform serial limb examinations every 1-2 hours while in the ED and admit for extended monitoring (24-48 hours) if there is a high index of suspicion, such as pain out of proportion, pain with passive stretch, or neurovascular changes. If the physical examination is unreliable, measure compartment pressure; an intracompartmental pressure >30 mmHg warrants immediate surgical consultation [31].
Perform a thorough musculoskeletal examination on all patients, as injuries may occur without visible entry or exit wounds [31].
Obtain plain radiographs for focal pain, swelling, decreased range of motion, or concern for fracture or dislocation [40,42,43].
Consult surgery for suspected compartment syndrome, fractures, dislocations, or high-voltage injuries with concern for deep tissue involvement; perform fasciotomy when indicated [31,34].
Perform prompt reduction of dislocations [43].

Renal

Electrical injuries often cause minimal superficial burns but more extensive muscle and deep tissue damage, resulting in tissue breakdown and rhabdomyolysis. Muscle breakdown leads to the release of myoglobin into the bloodstream, which can result in AKI. AKI in burn patients has been associated with increased mortality and length of hospital stay; therefore, aggressive fluid resuscitation is critical in this patient population [46].

AKI is defined by the Kidney Disease: Improving Global Outcomes (KDIGO) guidelines as an acute reduction in kidney function indicated by a urine output (UOP) of less than 0.5 mL/kg/hr for at least 6 hours, an increase in serum creatinine by 0.3 mg/dL within 48 hours, or a 1.5- to 2-fold increase from baseline [47]. Risk factors for AKI include severe thermal burns, HVEI, older age, and the presence of multiorgan failure [48]. AKI can present with dark red or brown urine, decreased frequency of urination, symptoms of volume overload such as pulmonary edema, dyspnea, and hypertension, and uremic symptoms such as nausea and altered mental status [49]. Serum creatinine is the primary marker of kidney function used for monitoring the development of AKI and should be monitored every 24 hours in most patients, or every 12 hours in patients at higher risk of developing AKI, such as those with extensive thermal burns, early evidence of rhabdomyolysis, and hemodynamic instability.

Intravenous fluid (IVF) resuscitation should be started immediately for those at increased risk of AKI and continued until 24 hours after electrical injury in order to prevent tissue hypoperfusion, AKI, and metabolic acidosis. Lactated Ringer’s is the preferred crystalloid fluid for resuscitation [50]. Several formulas exist to determine the volume of IVFs that should be given to these patients, including the Parkland, Brooke, and modified Brooke formulas. The Parkland formula states that 4 mL × total body surface area (TBSA) (%) × body weight (kg) is the total amount of IVFs required for a patient within the first 24 hours after sustaining a burn, with half of the total amount administered over the first 8 hours and the remaining half over the next 16 hours [51]. The Brooke formula uses 2 mL instead and combines crystalloid with colloid fluids. The modified Brooke formula recommends 2 mL × TBSA (%) × body weight (kg) of crystalloid in an effort to prevent over-resuscitation in these patients [52,53]. While the Parkland and modified Brooke formulas are the two most widely used, regardless of the formula utilized, it is recommended to start with one formula and then make adjustments based on UOP [53]. Of note, TBSA can be difficult to calculate in this patient population, as most of the tissue damage is internal, and the TBSA calculation is based solely on second- and third-degree burns using the Rule of Nines or the palmar surface method [54]. Because TBSA may underestimate injury severity in electrical burns, IVF resuscitation should be guided by UOP goals of 0.5-1 mL/kg/hr in adults with thermal burns, while adult patients with electrical injuries should have a target UOP of approximately 1 mL/kg/hr, which should be closely monitored during the hospital stay [51].

While IVF resuscitation is critical to preventing AKI, metabolic acidosis, and tissue hypoperfusion in burn patients, caution should be taken to avoid over-resuscitating these patients, as the complications of fluid overload can be just as harmful. Excessive fluid administration without adequate urine excretion can predispose patients to pulmonary, abdominal, and extremity edema, increasing the risk of respiratory complications and compartment syndromes [51,55,56]. Some proposed methods to prevent fluid overload include using the modified Brooke formula or relying on UOP as the primary marker of adequate fluid resuscitation.

An adjunctive therapy that has been proposed is the use of colloid fluids such as albumin or fresh frozen plasma (FFP) to help maintain oncotic pressure, which is lost because of the extensive endothelial and glycocalyx damage caused by burns; this strategy may reduce the risk of pulmonary and peripheral edema from resuscitation efforts. According to the ABRUPT study, albumin has been shown to be beneficial in reducing total IV fluid volume requirements in patients who were older, had inhalation injuries, showed signs of organ dysfunction, or sustained more severe burns involving greater than 20% TBSA that were unresponsive to crystalloid fluids [50]. It is recommended to start 5% albumin within 12 hours of the initial burn in this patient population, as it decreases morbidity risk [50,57]. Similarly, FFP has also been shown to reduce overall fluid requirements as well as lessen the inflammatory response that leads to burn shock and organ injury in burn patients [55,58]. Another study found that the use of FFP in burn patients reduced the risk of developing sepsis, renal failure, and acute respiratory distress syndrome (ARDS) [59].

AKI following electrical insults is mostly secondary to rhabdomyolysis rather than a primary insult to the kidneys themselves. AKI, regardless of its cause, is associated with a high rate of mortality and doubles the length of stay in the ICU; therefore, monitoring and prevention are essential (Table 3) [60]. It is recommended to obtain serum creatinine every 24 hours for the standard patient, and every 12 hours for those at higher risk of AKI development, along with continuous monitoring of UOP. IVF resuscitation is the cornerstone of AKI prevention and is essential for adequate tissue perfusion in burn patients. This is especially true in electrical burn patients, as most of the damage is to deep tissue and musculature and is therefore often underestimated. The Parkland and modified Brooke formulas are the most widely used protocols for fluid resuscitation; however, the primary goal should be UOP between 0.5-1 mL/kg/hr in adults with thermal burns and approximately 1 mL/kg/hr in adults with electrical injuries in order to prevent complications from over-resuscitation. The addition of FFP or albumin reduces volume requirements and can be incorporated into fluid resuscitation efforts in patients with severe burns or higher fluid rate requirements.

**Table 3:** Management of renal complications in the ED. Table Credits: Ireland Smith, Savannah Kidd IVF: Intravenous fluid; TBSA: Total body surface area; kg: Kilograms; mL: Milliliters; HVEI: High-voltage electrical injury; hr: Hour; UOP: Urine output; FFP: Fresh frozen plasma.

Management of renal complications
Immediately start IV fluid resuscitation with Lactated Ringer’s, following the Parkland formula (total amount of IVF = 4 mL × TBSA (%) × body weight (kg)) or the modified Brooke formula (total amount of IVF = 2 mL × TBSA (%) × body weight (kg)) in patients with TBSA >20% and/or HVEI/lightning exposure. Give one-half in the first 8 hours and the remainder in the next 16 hours [50-53].
Target UOP should be 1 mL/kg/hr for electrical injuries and 0.5-1 mL/kg/hr for thermal injuries [51].
Monitor for signs of over-resuscitation [51,55,56].
Five percent albumin or FFP may be added to fluid resuscitation efforts if the patient has sustained severe burns involving >40% TBSA or has fluid requirements above the standard 4 mL/kg/% TBSA rate [50,55,57,58].

Respiratory

An acutely fatal respiratory complication of electrical injury is respiratory arrest caused by paralysis or tetany of the diaphragm and intercostal muscles, or paralysis of the central respiratory centers from the electrical insult. Respiratory arrest is more commonly seen in lightning strike and HVEI victims because of the higher current of electricity, but it can also occur with LVEI depending on the trajectory of the electrical current [10]. Inhalation injuries can occur in electrical burn victims, especially if there are reports of fire, explosions, or the presence of combustible materials, gases, or toxins that may have been inhaled [45]. The upper airway is more susceptible to steam and hot gases, and injury can result in edema and airway obstruction. The trachea and bronchi are more often injured by chemical burns, resulting in bronchorrhea and bronchospasm [61,62].

Electrical insult can damage the lung parenchyma, resulting in pulmonary hemorrhage hours to days later, with dyspnea, hypoxia, and respiratory distress [63,64]. One case report described a patient who sustained an LVEI and presented with dyspnea and hemoptysis soon after the insult; imaging showed bilateral patchy infiltrates and consolidation in the lungs, indicative of acute pulmonary hemorrhage, which resolved after a week of treatment [64].

Respiratory arrest immediately after electrical injury should be treated according to ACLS protocol, initially with bag-valve-mask ventilation and ultimately endotracheal intubation to secure the airway if spontaneous respirations fail to occur [10]. If inhalation injury is suspected, then a physical examination, including examination of the nostrils, posterior pharynx, and lungs, should be performed as soon as possible by the emergency physician. If the patient has significant facial burns, singed nostril hair, throat pain, hoarseness, or erythema and ulceration of the pharynx, then direct laryngeal visualization is essential, either by fiberoptic bronchoscopy or during laryngoscopy. If there is high suspicion of present or impending airway obstruction, then endotracheal intubation should be performed immediately [61,62]. Delayed sequence intubation with ketamine is the recommended sedative approach in these patients, as pharyngeal muscle tone can be maintained during airway evaluation. In addition, if there are extensive facial burns, it is not recommended to secure the endotracheal tube (ETT) with adhesive tape or fabric ties; instead, the ETT should be anchored to the teeth by an oral and maxillofacial surgeon (OMFS) [65]. Patients with inhalation injury in the absence of airway obstruction can be managed with humidified air, elevation of the head of the bed, and close monitoring. All patients with inhalation injuries should be monitored for at least 72 hours, as the risk of airway edema is greatest during this period [66].

Pulmonary hemorrhage and ARDS should be suspected in patients with new-onset hemoptysis or respiratory distress, especially in those who sustained HVEI [66,67]. For these patients, hemoglobin and hematocrit should be measured to determine whether blood transfusion is necessary. A chest X-ray (CXR) or chest CT scan should be obtained to determine the location and size of the pulmonary contusion, as well as the severity of bleeding. If the bleeding is unilateral, the patient can be placed in Trendelenburg position with the affected side facing downward [68]. If the patient is in significant respiratory distress, intubation should be performed to protect the airway. The ETT should be large enough (7.5-8.0 mm) to allow for future bronchoscopic assessment to visualize any potential site of bleeding that can be targeted [69-71]. If bleeding is uncontrolled, then right mainstem intubation can be performed. Some studies and case reports have shown earlier cessation of bleeding when tranexamic acid (TXA) is used [72]. TXA can be administered via inhalation at 250-500 mg TID, intravenously at 500 to 2000 mg over 10 to 20 minutes undiluted or diluted in normal saline (NS), or as 500 mg/5 mL injected directly through the bronchoscope [67,72,73].

Although less common, if there was any accompanying fire or smoke inhalation with the electrical injury, carbon monoxide (CO) and cyanide toxicity must be suspected. CO poisoning can present with headache, dizziness, and lethargy and is diagnosed by arterial or venous carboxyhemoglobin levels or co-oximetry showing decreased oxygen-carrying capacity. CO poisoning can be reversed with 100% oxygen for approximately 40 minutes to 1 hour [61]. Cyanide poisoning is more common in structural fires because the burning of synthetic plastics releases hydrogen cyanide gas and can present with anion gap metabolic acidosis despite adequate oxygenation, as well as a significantly elevated plasma lactate concentration [74,75]. Treatment of cyanide toxicity requires administration of hydroxocobalamin (B12) and thiosulfate to reverse its effects [61]. A summary of the management of respiratory complications can be found in Table 4.

**Table 4:** Management of respiratory complications in the ED. Table Credits: Ireland Smith, Savannah Kidd ACLS: Advanced cardiac life support; OMFS: Oral and maxillofacial surgery; ETT: Endotracheal tube; CXR: Chest x-ray; Hgb: Hemoglobin; TXA: Tranexamic acid; CO,: Carbon monoxide.

Management of respiratory complications
Follow ACLS for respiratory arrest [10].
If inhalation injury is suspected: (1) perform a physical examination of the nostrils, posterior pharynx, and lungs; (2) perform direct laryngeal visualization or fiberoptic bronchoscopy for staging; (3) perform early intubation if there is concern for airway obstruction; (4) use delayed sequence intubation with ketamine as the preferred sedative approach; and (5) if facial burns are present, consult OMFS for tooth anchoring of the ETT [61,62,65].
Monitor patients with inhalation injuries for signs of delayed respiratory complications for at least 72 hours [66].
If pulmonary hemorrhage is suspected: (1) obtain hemoglobin, hematocrit, CXR, and/or chest CT; (2) transfuse if Hgb is <7; (3) if there is severe respiratory distress, intubate early to secure the airway; (4) for unilateral hemorrhage, place the patient with the affected side down, with or without Trendelenburg positioning, and consider right mainstem intubation; and (5) in severe hemorrhage, administer TXA [67-73].
If there has been close exposure to smoke or fumes, obtain a blood gas or co-oximetry on arrival at the ED to assess for CO poisoning. If CO is elevated, administer 100% oxygen. Suspected cyanide toxicity should be treated with thiosulfate and hydroxocobalamin (vitamin B12) [61].

Neurologic and Sensory

Neurologic injury in electrical burns occurs via thermal and non-thermal mechanisms. Thermal injury results from Joule heating, producing coagulation necrosis of neurons and supporting structures. Non-thermal mechanisms include electroporation, electroconformational changes in proteins, and direct current-induced axonal injury and demyelination [20,67]. Vascular spasm, thrombosis, and oxidative stress further contribute to ischemia, infarction, and delayed progressive deficits [76-78].

Electrical injuries can result in both acute and delayed neurologic deficits involving the central and peripheral nervous systems (PNSs). Acute CNS manifestations include loss of consciousness, seizures, abnormal involuntary movements, ischemic or hemorrhagic stroke, cerebellar ataxia, and hypoxic encephalopathy. PNS involvement may present with sensory disturbances, mononeuropathies, plexopathies, muscle weakness, or transient paralysis [36,76,79]. In some cases, intense nerve stimulation may trigger massive muscle contraction, leading to secondary trauma such as fractures or dislocations [36]. Delayed neurologic sequelae may emerge days to years after the injury and often progress insidiously. CNS complications in this phase include epilepsy, cognitive dysfunction, headaches, vertigo, and abnormal movements [78-80]. Chronic PNS complications may manifest as neuropathy, persistent sensory deficits, plexopathies, or chronic pain [77,79,81].

Evaluation should begin with a comprehensive neurologic examination and continuous monitoring for evolving deficits. Neuroimaging with CT or MRI is indicated when focal findings, altered mental status, or suspicion for stroke or hemorrhage is present [79]. EEG can also be useful in patients with seizures or unexplained altered consciousness [82]. Although no laboratory test directly identifies neurologic injury, baseline studies are recommended to screen for systemic complications that may influence neurologic status. Serum CK helps detect rhabdomyolysis, which is common in deep muscle injury and may contribute to compartment syndrome or secondary nerve damage. Renal function tests (BUN, creatinine) and electrolytes (especially potassium) should be obtained to identify AKI and metabolic derangements that can exacerbate neurologic symptoms or complicate management [77,83,84]. For patients with peripheral neuropathies, outpatient neurology referral is appropriate [85-87].

Although electrical injury increases the risk of epilepsy, the overall incidence remains low [79]. Routine seizure prophylaxis is not recommended. Antiepileptic drugs should be reserved for patients with high-risk features such as intracranial injury, prolonged loss of consciousness, or documented seizures [88].

Electrical burns can also affect sensory organs. Vestibulocochlear injury may cause dizziness, tinnitus, or imbalance, warranting a full otologic evaluation in patients with head or ear involvement [89]. Tympanic membrane rupture, a hallmark of lightning injury, is less common in domestic or industrial burns [90]. In cases of tympanic membrane injury, early otolaryngology consultation is recommended to determine the need for surgical repair.

Ophthalmologic complications range from acute corneal erosions and anterior segment burns to late sequelae such as cataract formation or macular cysts, which may cause progressive vision loss. Initial visual symptoms may include visual disturbances, pain, photophobia, and decreased visual acuity, with corneal erosions being the most common cause of visual symptoms [91,92]. Early ophthalmology consultation is crucial for patients with facial burns or visual symptoms. Initial management includes irrigation when contamination is suspected, followed by lubricants, topical antibiotics, and fluorescein staining under a Wood’s lamp to detect corneal injury [91,93]. In severe cases, amniotic membrane transplantation or surgical intervention may be required to protect vision [94,95].

Pain management is another critical component of care. Initial management of electrical injuries should employ a multimodal approach that addresses both nociceptive and neuropathic pain components. Burn-related pain may be acute, neuropathic, or secondary to trauma. Mild to moderate pain can be managed with acetaminophen and nonsteroidal anti-inflammatory drugs (NSAIDs), while opioids are reserved for moderate to severe pain. Neuropathic pain often requires adjuvant therapy with gabapentin or pregabalin, although these may increase dizziness in patients with vestibular symptoms. For refractory pain, ketamine or alpha-2 agonists such as dexmedetomidine and clonidine may be used [96]. A summary of the management of neurologic and sensory complications can be found in Table 5.

**Table 5:** Management of neurologic and sensory complications in the ED. Table Credits: Ireland Smith, Savannah Kidd NSAID: Nonsteroidal anti-inflammatory drug; CK: Creatine kinase; BUN: Blood urea nitrogen.

Management of Neurological & Sensory Complications
Perform thorough neurologic exam; obtain CT/MRI if focal CNS findings, altered mental status, or loss of consciousness [79]
EEG for seizures or unexplained altered consciousness [82]
Evaluate vestibulocochlear and ocular involvement in all head/face burns [89-92]
Initiate early ophthalmology or otolaryngology consultation if symptoms present [89,91,93]
Manage pain with a multimodal, stepwise approach: acetaminophen/NSAIDs for mild-moderate pain → opioids for moderate-severe pain. Add adjuvant therapies (gabapentinoids, ketamine, or alpha-2 agonists) as needed for neuropathic or refractory pain [96]
Avoid routine seizure prophylaxis, unless patient is high-risk [88]
Labs: CK, BUN/Creatinine, and electrolytes to monitor for rhabdomyolysis, kidney injury, and metabolic complications that could affect neurologic status [77,83,84]

Cutaneous

Cutaneous wounds may appear deceptively minor compared with the severity of the underlying tissue injury. Electrical current travels preferentially through low-resistance tissues such as muscle and neurovascular bundles, resulting in deep myonecrosis, while the skin itself functions as both a resistor and a conductor [35,97]. Cutaneous injury arises from both direct thermal damage due to resistance heating and non-thermal mechanisms, such as electroporation, which disrupts cell membranes and causes cell death. High-voltage injuries often produce coagulation necrosis at entry and exit sites, with deep tissue injury along the current pathway, frequently sparing the intervening skin. Thus, visible burns do not reliably reflect the true extent of injury [10,35,97].

Burns are classified by their depth. Superficial burns (first-degree) involve only the epidermis and present as erythematous and painful. Partial-thickness burns (second-degree) extend into the dermis: superficial partial-thickness burns blister and blanch, whereas deeper partial-thickness burns are paler, less blanchable, and more painful. Full-thickness burns (third-degree) destroy the entire epidermis and dermis, appear leathery or charred, are insensate, and require surgical management [50,98]. Circumferential full-thickness burns may impair perfusion or precipitate compartment syndrome, requiring escharotomy [99].

When assessing burn severity, the TBSA should be estimated for partial- and full-thickness burns (superficial burns are excluded). The Rule of Nines is the standard clinical tool, assigning percentages to anatomic regions: each arm 9%, each leg 18%, anterior trunk 18%, posterior trunk 18%, head/neck 9%, and perineum 1% [50]. Although widely used, it may be inaccurate in obese patients or those with noncontiguous burn patterns, often overestimating TBSA. Newer technology-based models, such as EasyTBSA and Burn Area, improve accuracy but are not yet universally adopted [100-102].

Typical features of electrical burns include small, well-demarcated entry and exit wounds with a dry, leathery, or charred appearance, surrounded by pallor or erythema. Upper extremities are often affected because of hand contact, with exit wounds on the contralateral hand or foot [35,97]. Characteristic patterns include “crocodile skin” (tightly spaced round burns), metallization (conductor vapor deposition), and periorbital “crow’s feet” burns from facial arcs [103]. Lightning injuries, a subtype of electrical burn, may present with transient Lichtenberg figures, fern-like erythematous skin markings that resolve within 24 hours. Other lightning-associated patterns include linear burns from vaporized sweat, punctate exit-site burns, and singed body hair. Lightning strikes usually cause less severe skin injury than high-voltage electrical burns, as the current is brief and tends to travel over the skin surface rather than deeply [6,104].

Management is guided by burn depth and systemic impact. Superficial burns are managed with gentle cleansing, moist dressings, and topical antimicrobials such as bacitracin, neomycin, or polymyxin [50]. Full-thickness burns may require silver sulfadiazine (avoided on the face), chemical debridement, or surgical excision [105]. Early consultation with a burn center or burn surgery team is recommended for all electrical burns, given the high likelihood of deep tissue involvement and the need for operative intervention [99,106]. Escharotomy is urgent in circumferential full-thickness burns with evidence of vascular compromise or impending compartment syndrome. Clinical signs include decreased or absent distal pulses, increasing pain, pallor, paresthesia, paralysis, poikilothermia, and swelling. Intracompartment pressures greater than 30 mm Hg, delta pressures (diastolic blood pressure, intracompartment pressure) less than 30 mm Hg, or absent Doppler signals confirm the need for urgent escharotomy [34,99,107].

Cutaneous injury assessment in electrical burns requires careful inspection for entry and exit wounds, burn depth and TBSA assessment, and recognition that visible skin injury underestimates deeper damage. Early burn specialist involvement is essential. A summary of the management of cutaneous complications in the emergency department is provided in Table 6.

**Table 6:** Management of cutaneous complications in the ED. Table Credits: Ireland Smith, Savannah Kidd TBSA: Total Body Surface Area.

Management of cutaneous complications
Carefully inspect for wounds that may represent entry and exit sites, as well as characteristic burn patterns [6,35,97,103,104].
Calculate TBSA for fluid resuscitation, while recognizing its limitations [51].
Cleanse superficial wounds gently; apply moist dressings and topical antimicrobials [50].
Full-thickness burns may require silver sulfadiazine (avoided on the face), chemical debridement, or surgical excision, with burn team consultation [105].
Monitor for vascular compromise; perform urgent escharotomy if indicated [34,99,106].
Consult burn specialists for all electrical burns, regardless of cutaneous appearance, given the high risk of deeper tissue involvement [99,106].

Disposition

Disposition for patients with electrical injuries depends on a variety of factors and ultimately comes down to the initial presentation of the individual patient. Some factors include the voltage of electricity that the patient came into contact with, the patient’s initial physiological response to the electricity (i.e., arrest, arrhythmia, airway edema, etc.), laboratory markers, and the potential for complications within the first 24 to 72 hours. HVEI from occupational hazards and lightning strikes are more often associated with serious complications such as cardiac arrest, respiratory arrest, inhalation injuries, compartment syndrome, AKI, and seizures. Therefore, these patients should be admitted, often to burn units or ICUs, as they frequently require emergent interventions, fluid resuscitation, and prolonged monitoring by medical professionals.

Indications for admission include, but are not limited to, cardiac arrest, abnormal ECG on arrival to the ED, loss of consciousness from the electrical shock, hemodynamic instability, respiratory arrest, the presence of inhalation injuries, signs of pulmonary hemorrhage, CK greater than 5 times normal, AKI, receipt of fluid resuscitation, the need for IV pain management, the presence of partial- or full-thickness burns, circumferential burns, and signs of compartment syndrome. In order to adequately treat these conditions and ensure patient safety, these patients should be admitted to the hospital for monitoring and management of symptoms.

Patients who have sustained LVEI are often considered to be at lower risk of developing serious life-threatening complications requiring acute medical intervention than those who have sustained HVEI or lightning injuries. Likewise, patients who have normal ECGs upon arrival to the ED, no loss of consciousness or arrest of any kind at the scene, no thermal burns and therefore no need for fluid resuscitation, normal CK, normal baseline creatinine, and no need for continued IV pain management can most likely be discharged from the ED with appropriate follow-up with their primary care physicians to ensure monitoring for long-term, non-emergent complications.

**Figure 1:**
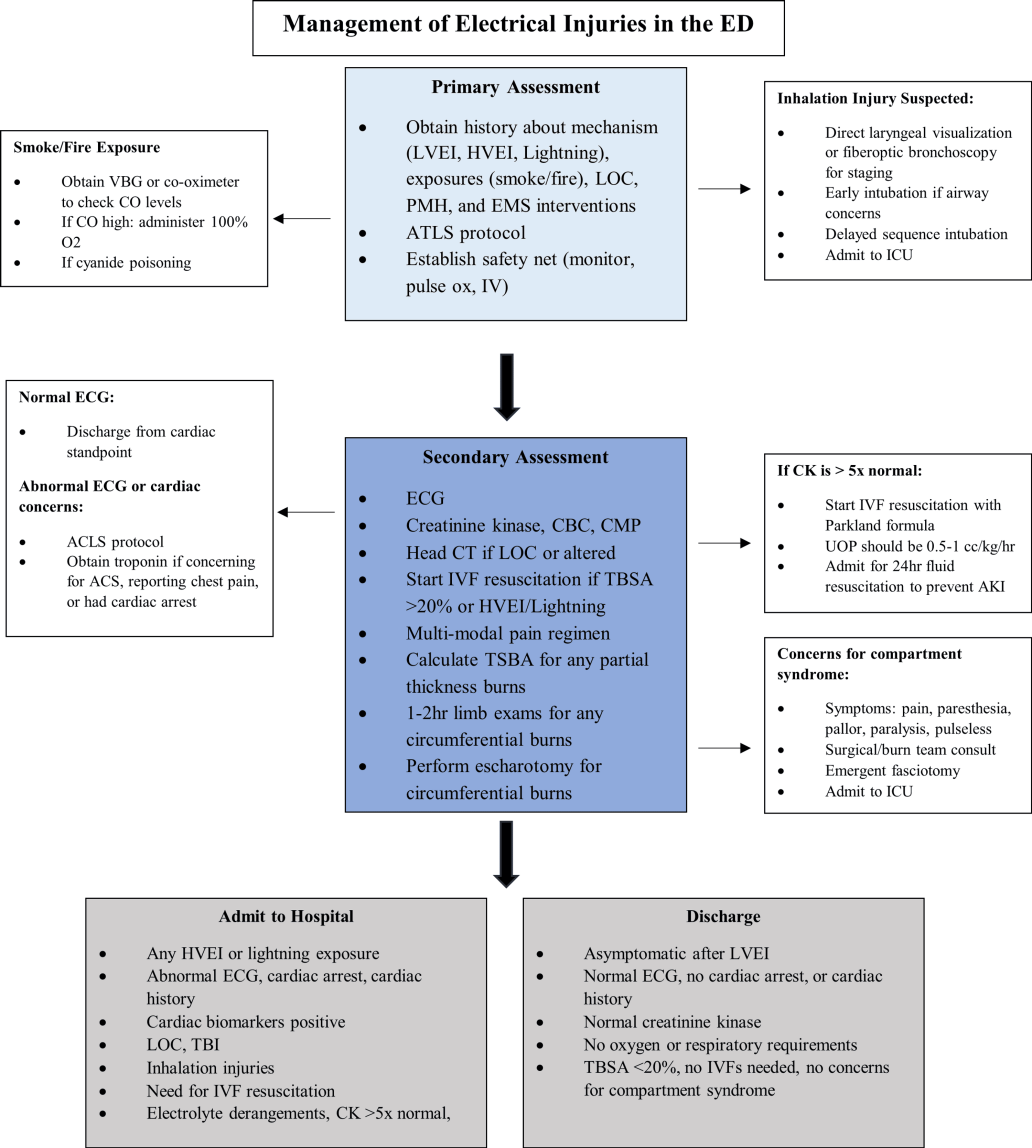
Flow chart of management of electrical injuries in the ED. Figure Credits: Ireland Smith, Savannah Kidd ACLS: Advanced cardiac life support; ACS: Acute coronary syndrome; AKI: Acute kidney injury; ATLS: Advanced trauma life support; CBC: Complete blood count; CMP: Comprehensive metabolic panel; CO: Carbon monoxide; EMS: Emergency medical services; HVEI: High-voltage electrical injury; IVF: Intravenous fluids; LOC: Loss of consciousness; LVEI: Low-voltage electrical injury; O₂: Oxygen; PMH: Past medical history; TBSA: Total body surface area; UOP: Urine output; VBG: Venous blood gas.

**Figure 2:**
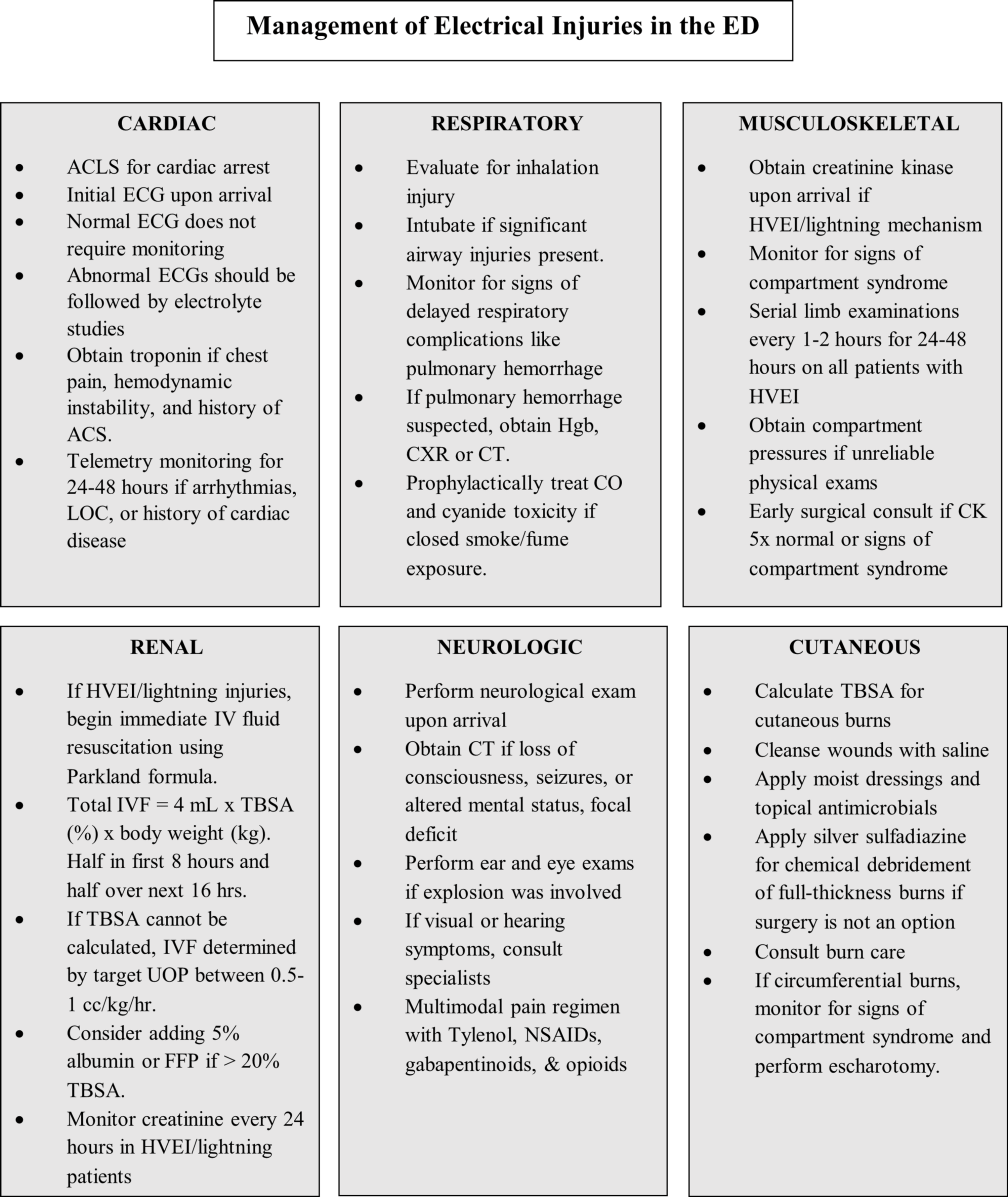
Management of electrical injuries by organ system in the ED. Figure Credits: Ireland Smith, Savannah Kidd ACLS: Advanced cardiac life support; ACS: Acute coronary syndrome; CK: Creatine kinase; CO: Carbon monoxide; CXR: Chest x-ray; FFP: Fresh frozen plasma; HVEI: High-voltage electrical injury; IVF: Intravenous fluids; LOC: Loss of consciousness; LVEI: Low-voltage electrical injury; NSAIDs: Nonsteroidal anti-inflammatory drugs; TBSA: Total body surface area; UOP: Urine output.

Proposed structured approaches are summarized in Figure 1, which outlines assessment of electrical injuries in the ED, and Figure 2, which highlights organ system-based management strategies.

## Conclusions

Electrical burn injuries pose a unique diagnostic and management challenge, often causing systemic injury that may not be apparent on initial examination. Currently, there are no standard guidelines for the assessment and management of electrical injuries beyond lightning strikes, as most are managed with a combination of trauma and burn protocols. Based on the current literature, rapid trauma assessment accompanied by assessment of the ABCs, CBC, CMP, ECG, and CK monitoring, fluid resuscitation when indicated, and a multidisciplinary team approach are recommended for this patient population. For emergency providers, recognizing subtle presentations and having structured protocols in place can help reduce delays, minimize missed complications, and improve survival. Moving forward, the development of standardized ED protocols and improved tools for risk stratification will be critical to advancing care and outcomes in this high-risk population.
